# Does adding a delayed phase to cardiac computed tomography for coronary artery evaluation have prognostic value?

**DOI:** 10.1093/ehjci/jeag018

**Published:** 2026-01-22

**Authors:** Tetsuya Oguni, Yasuhiro Izumiya, Seitaro Oda, Seij Takashio, Yosuke Matsumoto, Naoto Kuyama, Shinsuke Hanatani, Hiroki Usuku, Yasushi Matsuzawa, Masafumi Kidoh, Eiichiro Yamamoto, Toshinori Hirai, Kenichi Tsujita

**Affiliations:** Department of Cardiovascular Medicine, Graduate School of Medical Sciences, Kumamoto University, 1-1-1 Honjo, Chuo-ku, Kumamoto 860-8556, Japan; Department of Cardiovascular Medicine, Graduate School of Medical Sciences, Kumamoto University, 1-1-1 Honjo, Chuo-ku, Kumamoto 860-8556, Japan; Department of Diagnostic Radiology, Graduate School of Medical Sciences, Kumamoto University, 1-1-1 Honjo, Chuo-ku, Kumamoto 860-8556, Japan; Department of Cardiovascular Medicine, Graduate School of Medical Sciences, Kumamoto University, 1-1-1 Honjo, Chuo-ku, Kumamoto 860-8556, Japan; Department of Cardiovascular Medicine, Graduate School of Medical Sciences, Kumamoto University, 1-1-1 Honjo, Chuo-ku, Kumamoto 860-8556, Japan; Department of Cardiovascular Medicine, Graduate School of Medical Sciences, Kumamoto University, 1-1-1 Honjo, Chuo-ku, Kumamoto 860-8556, Japan; Department of Cardiovascular Medicine, Graduate School of Medical Sciences, Kumamoto University, 1-1-1 Honjo, Chuo-ku, Kumamoto 860-8556, Japan; Department of Cardiovascular Medicine, Graduate School of Medical Sciences, Kumamoto University, 1-1-1 Honjo, Chuo-ku, Kumamoto 860-8556, Japan; Department of Cardiovascular Medicine, Graduate School of Medical Sciences, Kumamoto University, 1-1-1 Honjo, Chuo-ku, Kumamoto 860-8556, Japan; Department of Diagnostic Radiology, Graduate School of Medical Sciences, Kumamoto University, 1-1-1 Honjo, Chuo-ku, Kumamoto 860-8556, Japan; Department of Cardiovascular Medicine, Graduate School of Medical Sciences, Kumamoto University, 1-1-1 Honjo, Chuo-ku, Kumamoto 860-8556, Japan; Department of Diagnostic Radiology, Graduate School of Medical Sciences, Kumamoto University, 1-1-1 Honjo, Chuo-ku, Kumamoto 860-8556, Japan; Department of Cardiovascular Medicine, Graduate School of Medical Sciences, Kumamoto University, 1-1-1 Honjo, Chuo-ku, Kumamoto 860-8556, Japan; Center of Metabolic Regulation of Healthy Aging, Faculty of Life Sciences, Kumamoto University, 1-1-1 Honjo, Chuo-ku, Kumamoto 860-8556, Japan

**Keywords:** cardiovascular event, coronary artery, myocardium

## Abstract

**Aims:**

Cardiac computed tomography (CCT) assesses coronary anatomy and enables delayed-phase imaging, including extracellular volume fraction (ECV) for diffuse myocardial fibrosis and late iodine enhancement (LIE) for focal myocardial replacement fibrosis. ECV and LIE reflect distinct pathological processes; combining these measures may improve subclinical myocardial injury detection. This study evaluated LIE and ECV in patients undergoing CCT for coronary artery assessment and examined their association with clinical outcomes. The primary outcome was a composite of all-cause death and unplanned cardiovascular hospitalizations; the secondary outcome was cardiovascular events, defined as cardiac death and unplanned cardiovascular hospitalization.

**Methods and results:**

We analysed 1207 consecutive patients who underwent CCT between January 2020 and September 2022. Patients were categorized into four groups based on the presence of LIE and elevated ECV. Associations with LIE and ECV, individually and combined, were assessed using Cox proportional hazards models. Of 1305 patients, 1207 met inclusion criteria and were followed for a mean of 26.0 ± 19.1 months. Kaplan–Meier analysis demonstrated a stepwise increase in risk across the four groups, with those having LIE and elevated ECV showing the highest cumulative incidence of composite events (log-rank *P* = 0.027). This group had increased risk for the composite outcome [hazard ratio (HR) 1.84, 95% confidence interval (CI) 1.22–2.79] and cardiovascular events (HR 2.67, 95% CI 1.32–5.41).

**Conclusion:**

In patients undergoing CCT for coronary artery evaluation, coexistence of LIE and elevated ECV is associated with higher risk of cardiovascular events and their assessment may provide synergistic prognostic value.

## Graphical Abstract

**Graphical Abstract:**
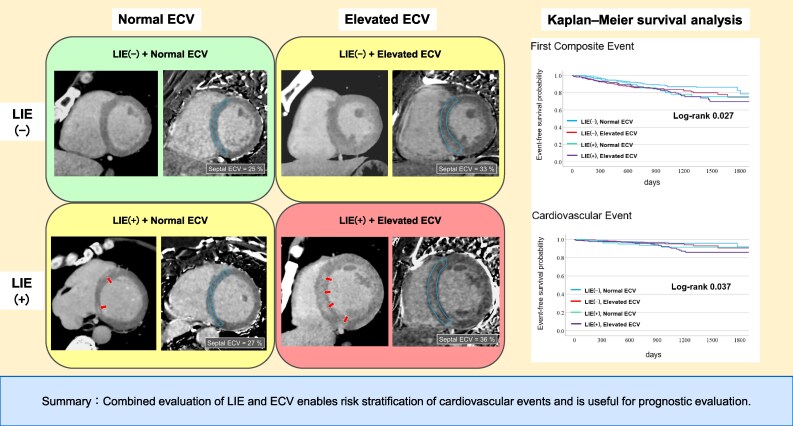

**See the editorial comment for this article ‘Delayed CT imaging in coronary artery disease: ready for prime time?’, by L. Slipczuk**  ***et al*****., https://doi.org/10.1093/ehjci/jeag053.**

## Introduction

In recent years, non-invasive cardiac imaging has become essential for diagnosing, characterizing, and risk-stratifying a broad range of cardiovascular diseases, including ischaemic and non-ischaemic cardiomyopathies.^1^ Cardiac magnetic resonance (CMR) remains the reference standard for myocardial tissue characterization due to its capacity to assess focal and diffuse myocardial injury.^2,3^ However, recent advancements in cardiac computed tomography (CCT) have expanded its role beyond anatomical imaging, enabling tissue characterization comparable to that with CMR.^4^

Cardiac computed tomography angiography (CCTA) is widely used for evaluating coronary artery disease (CAD) because of its high diagnostic accuracy and excellent negative predictive value.^5^ More recently, delayed-phase imaging techniques—such as late iodine enhancement (LIE) and computed tomography–derived extracellular volume fraction (CT-ECV)—have been developed to assess myocardial injury within the same session as CCTA.^6^ Multiple studies have demonstrated strong agreement between CT-ECV and CMR-derived ECV, as well as between LIE and late gadolinium enhancement (LGE), supporting CCT as a potential alternative to CMR for myocardial tissue characterization.^7,8^ Furthermore, CT-ECV has shown prognostic value in patients undergoing transcatheter aortic valve implantation (TAVI) and in those with infiltrative cardiomyopathies, such as cardiac amyloidosis, where it correlates with adverse myocardial remodelling and all-cause mortality.^9–11^

Although ECV and LIE can be obtained from delayed-phase CCT, they represent distinct pathological substrates. ECV offers a quantitative measure of diffuse interstitial expansion, whereas LIE identifies localized fibrosis or necrosis.^12–14^ Each provides complementary insights into myocardial health and disease progression. Combining CT-ECV and LIE may therefore improve detection of subclinical myocardial injury and enhance prediction of cardiovascular events.

While the combined assessment of LIE and ECV has been proposed to add prognostic value beyond traditional coronary artery anatomical evaluation, particularly for risk stratification, its prognostic significance remains unclear in patients undergoing CCT for CAD evaluation.^15^ This study aimed to determine the prognostic impact of simultaneous LIE and ECV assessment in patients undergoing CCTA and evaluate their relationship with cardiovascular events.

## Methods

### Study design and ethical considerations

This single-centre retrospective study was conducted in accordance with the Declaration of Helsinki and approved by the Institutional Review Board and Ethics Committee of Kumamoto University (approval no. 2384).

### Consent for participation

Given the retrospective design and minimal risk to participants, informed consent was waived. Consistent with institutional policy, the study protocol was posted on the Kumamoto University Hospital website (https://kumadai-radiology.jp/medical-research/), allowing patients the opportunity to opt out.

### Study population

Clinical records of patients with suspected coronary artery disease who underwent CCTA for clinical indications based on European Society of Cardiology guidelines between January 2020 and September 2022 were retrospectively reviewed. No additional clinical conditions, such as a history of myocardial infarction or persistent atrial fibrillation, were applied as exclusion criteria beyond those explicitly defined. Patients with mild or moderate aortic stenosis (AS) were included; however, those with severe AS—defined as an aortic valve area (AVA) ≤ 1.0 cm^2^ (or an indexed AVA ≤0.6 cm^2^/m^2^), mean gradient ≥40 mmHg, and peak velocity ≥4.0 m/s—were excluded because, at our institution, such patients routinely undergo a dedicated TAVI-planning CT protocol that differs from the standard CCT protocol used in this study.^16^ Exclusion criteria included: (i) absence of LIE or ECV assessment, (ii) suboptimal delayed enhancement image quality, and (iii) prior diagnosis of non-ischaemic cardiomyopathy (NICM), such as sarcoidosis, hypertrophic cardiomyopathy (HCM), cardiac amyloidosis, or dilated cardiomyopathy (DCM), before undergoing CCT.

### CCT imaging protocol

In our institution, delayed-phase cardiac CT (equilibrium-phase imaging) was incorporated into the routine clinical CCTA protocol to enable the assessment of LIE and calculation of the ECV. A 320 × 0.5 mm detector row CT unit (Aquilion One Genesis edition; Canon Medical Systems) was used for CT examinations. First, pre-contrast electrocardiogram-gated cardiac CT was performed at 120 kVp (0.275 ms/rot) in mid-diastole (75% of the R–R interval) at low heart rates or in end-systole (30–40% of the R–R interval) at high heart rates (>70 beats/min). Second, CCTA was performed at the optimal cardiac phase, matched to the pre-contrast scan. Finally, delayed-phase cardiac CT was acquired at 120 kVp (0.275 ms/rot) in the same cardiac phase as the pre-contrast scan. CCTA was performed using a bolus-tracking technique triggered at the ascending aorta, with intravenous injection of 450 mgI/kg of contrast medium over 15 s. To enhance visualization of LIE, an additional 100 mgI/kg of contrast medium was administered over 10 s after CCTA, followed by delayed-phase imaging 7 min later. In total, ∼550 mgI/kg of iodine (Iopamiron 370, Bayer Healthcare) was administered. To minimize the risk of nephrotoxicity, contrast medium use was tailored according to each patient’s renal function. For patients with chronic kidney disease and an estimated glomerular filtration rate (eGFR) of 30–45 mL/min/1.73 m², the dose was reduced by 20% to 450 mgI/kg. Contrast-enhanced CT was avoided in patients with eGFR <30 mL/min/1.73 m². A fixed high tube current (750 mA) was applied during delayed-phase imaging to achieve sufficient noise and artefact reduction for the visualization of late myocardial enhancement. In representative cases, the volume CT dose index (CTDI_vol_) for delayed-phase imaging was 10.6 mGy, and the dose length product (DLP) was 169 mGy cm. In our institution, the radiation exposure data for the routine cardiac CT protocol (non-contrast, CCTA, and delayed phase) showed median values of CTDIvol, DLP, and effective radiation dose of 44.0 mGy [interquartile range (IQR), 27.0–65.3], 504.4 mGy cm (IQR, 309.5–748.6), and 7.1 mSv (IQR, 4.4–10.5), respectively. The overall radiation dose of the CCT protocol, including delayed-phase cardiac CT, was considered sufficiently low to be justified in accordance with the ‘as low as reasonably achievable (ALARA)’ principle.

Artefacts typically appeared as areas of void/missing voxels. Where necessary, image co-registration was repeated and manually corrected to compensate for artefacts. In case of persistent poor image quality, datasets were excluded from analysis.

Significant CAD was defined as >70% stenosis (>50% only in the left main coronary artery) on cardiac CT.^17^

### Late contrast enhancement analysis

LIE images were analysed by two expert readers using a dedicated workstation application (Ziostation2; Ziosoft, Tokyo, Japan). LIE was visually defined as a localized area of hyperattenuation relative to surrounding myocardium in two orthogonal planes on post-contrast images,^18^ and evaluated according to the American Heart Association (AHA) 17-segment model. LIE patterns were classified as ischaemic (subendocardial or transmural) or non-ischaemic (subepicardial or intramyocardial), following LGE pattern criteria.^3^ Subendocardial LIE patterns consistent with coronary territory distribution were classified as ischaemic; those inconsistent with coronary territory distribution, as well as diffuse LIE, were classified as non-ischaemic. Patients with combined ischaemic and non-ischaemic patterns were included in the ischaemic group. Any patient with LIE in at least one of the 17 segments, regardless of ischaemic or non-ischaemic pattern, was assigned to the LIE group.

### CT-ECV analysis

**Figure 1:**
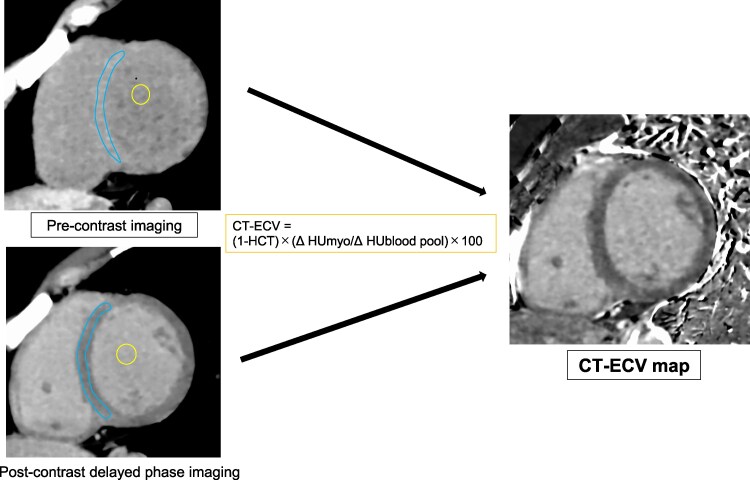
Extracellular volume analysis in CCT. The diagram shows the ECV measurement method. The CT-ECV is calculated as CT-ECV = (1 − haematocrit) × (Δmyo/Δblood pool) × 100. ROIs are drawn manually on the septal segment of the mid-ventricular short-axis multiplanar reconstruction images of the CT-ECV map. ECV, extracellular volume fraction; CT, computed tomography; HCT, haematocrit; Δmyo, myocardial Hounsfield units (post−pre-contrast); Δblood pool, left ventricular Hounsfield units (post−pre-contrast).

Myocardial CT-ECV quantification was performed on pre- and post-contrast delayed-phase CT images using the subtraction method. Pre- and post-contrast images were processed in Ziostation2 to generate CT-ECV maps. The myocardial CT-ECV was calculated as follows:


CT-ECV=(1−hematocrit)×(ΔHUmyo/ΔHUbloodpool)×100

where Δmyo represents the change in the myocardial Hounsfield units (post-contrast–pre-contrast) and Δblood pool is the change in the left ventricular Hounsfield units (post-contrast–pre-contrast). Regions of interest (ROIs) were manually drawn on the septal segment of mid-ventricular short-axis multiplanar reconstruction images of the CT-ECV map. ROIs were maximized to encompass the septal segment from the epicardium to the endocardium. Only myocardial pixels unaffected by the in-plane partial volume effect from blood, epicardial fat, or pericardial fluid were included. Myocardial segments with LIE in the left ventricular septum were also included for ECV measurement (*Figure 1*).

To achieve consensus, ECV measurements were performed by two expert readers (M.K. and S.O., with 14 and 18 years of experience, respectively), blinded to clinical information, similar to the evaluation of LIE images. In cases of disagreement between the two experts, the final decision was made by consensus after joint review and discussion.

### Biomarker analysis

Serum high-sensitivity cardiac troponin T (hs-cTnT; normal cut-off: 0.014 pg/mL) was measured at diagnosis using the Elecsys 2010 Troponin T hs kit (Roche Diagnostics, Indianapolis, IN, USA). Plasma B-type natriuretic peptide (BNP; normal cut-off: 18.4 pg/mL) was measured using the MI02 Shionogi BNP kit (Abbott Japan, Matsudo, Japan). hs-cTnT and BNP levels were obtained within 60 days of CT examination under stable conditions. The glomerular filtration rate (GFR) was calculated using the Modification of Diet in Renal Disease Study equation, modified for Japanese individuals.

### Echocardiographic analysis

Echocardiography was performed using Vivid E95 or 7 (GE Vingmed, Horten, Norway), Aplio 500 (Canon Medical Systems Corp., Otawara, Tochigi, Japan), or Epiq 7G (Philips, Bothell, WA, USA). Conventional echocardiography followed the recommendations of the American Society of Echocardiography and the European Association of Cardiovascular Imaging.^19,20^ The most recent echocardiographic data within 60 days of CT examination were used. Cardiac chamber size and wall thickness were measured in the transthoracic view, and left ventricular ejection fraction (LVEF) was calculated using the modified Simpson method.

### Clinical follow-up and endpoints

Patient follow-up was conducted through review of medical records, supplemented by questionnaires and direct telephone interviews with patients or, if deceased, their family members. Causes of death were determined through a collective review of death certificates, in-hospital medical records, and, when available, postmortem findings. The primary outcome was the earliest occurrence of either unplanned cardiovascular hospitalization or all-cause death after the baseline CCT scan. The secondary outcome was defined as cardiovascular events, including cardiac death incidence and unplanned cardiovascular hospitalization during follow-up. Cardiac death was defined as death from worsening heart failure (HF), acute coronary syndrome, or sudden death. Planned cardiovascular hospitalization referred to elective admissions scheduled for diagnostic or therapeutic cardiovascular procedures, such as percutaneous coronary intervention or device replacement. Unplanned cardiovascular hospitalizations were defined as admissions due to acute clinical events, specifically acute HF or acute coronary syndrome. Hospitalization for HF was defined as admission with signs and symptoms of decompensated HF requiring intravenous treatment (diuretics, vasodilators, or inotropes). Follow-up duration was calculated from the baseline CCT date and censored at the first event or last patient contact.

The clinical events were adjudicated by two independent board-certified cardiologists (T.O. and Y.I.).

### Statistical analysis

Statistical analyses were performed using IBM SPSS Statistics, version 29 (IBM Corporation, Armonk, NY, USA). Normally distributed variables are reported as mean ± standard deviation (SD), and non-normally distributed variables as median with IQR. Categorical variables are presented as numbers (percentages). We conducted a complete case analysis, excluding records with missing values for key covariates. The number and proportion of missing observations for each variable are reported in Supplementary data online, *Table S1*. Differences among the four groups were assessed using one-way analysis of variance or the Kruskal–Wallis test for continuous variables, and the *χ*^2^ test for categorical variables. *Post hoc* pairwise comparisons were performed using Tukey’s test or Dunn–Bonferroni correction for continuous variables, and using the *χ*^2^ test with Bonferroni correction for categorical variables, as appropriate. Kaplan–Meier survival analysis was used to compare all-cause death, cardiac death, and unplanned cardiovascular hospitalization between subgroups during follow-up. The log-rank test compared Kaplan–Meier curves. Multivariable analyses of event-free survival were conducted using Cox proportional hazards regression. Covariates were selected *a priori* based on prior literature and clinical experience, including age, sex, hypertension, diabetes, and coronary revascularization before CCT. In addition, *post hoc* covariates were determined based on the significance of their associations with the outcome in univariable Cox proportional hazards analyses. As a result, the LVEF was included as a *post hoc* covariate in the multivariable model, given its prognostic impact. All eligible variables were assessed for collinearity using the variance inflation factor before inclusion, with all final models showing low multicollinearity. Receiver operating characteristic (ROC) analysis with the Youden index was performed in the entire cohort to determine the optimal ECV cut-off value for predicting the first composite events during follow-up, which was identified as 29.5%. Statistical significance was set at a two-tailed *P*-value <0.05.

### Diagnosis of NICM by CCT

In cases where NICM was suspected on CCT, additional diagnostic evaluations, including CMR, endomyocardial biopsy, and 99mTc-PYP scintigraphy, were performed to confirm the diagnosis.

## Results

### Clinical characteristics of the patients

**Figure 2:**
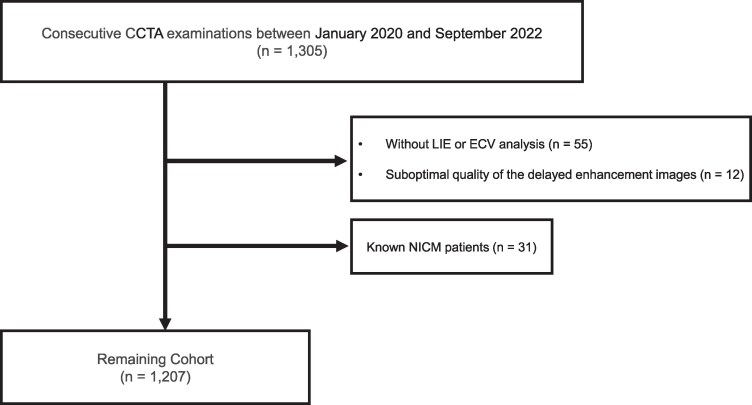
Study flow diagram illustrating the application of inclusion and exclusion criteria to the cohort. CCTA, cardiac computed tomography angiography; LIE, late iodine enhancement; NICM, non-ischaemic cardiomyopathy.

**Figure 3:**
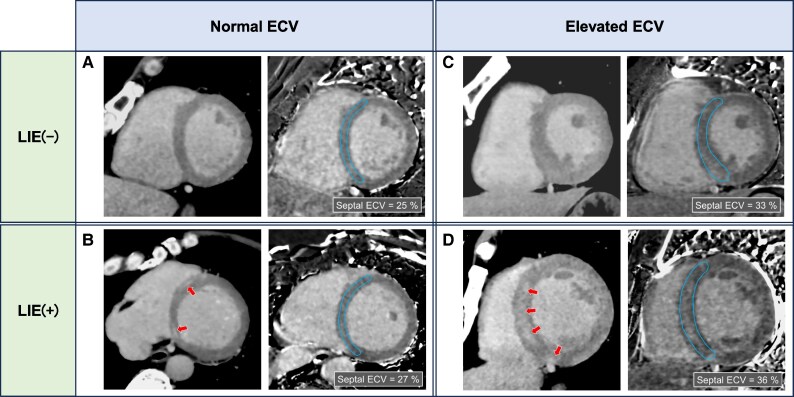
Representative CCT images according to LIE and ECV status. Representative CCT images from four groups: (*A*) no LIE with normal ECV, (*B*) LIE with normal ECV, (*C*) no LIE with elevated ECV, and (*D*) LIE with elevated ECV. Two examples per group demonstrate imaging differences by LIE and ECV patterns. Left panel: CT scan in the mid-cardiac short-axis view showing LIE at the arrow. Right panel: Corresponding CT-ECV maps with similar myocardial involvement. All images were evaluated in the mid-cardiac short-axis view. CCT, cardiac computed tomography; LIE, late iodine enhancement; ECV, extracellular volume fraction.

*Figure 2* illustrates the patient selection process. Of 1305 consecutive patients who underwent CCTA between January 2020 and September 2022, 1207 were included in the final analysis. Ninety-eight patients were excluded due to the absence of LIE or ECV analysis (*n* = 55), suboptimal delayed enhancement image quality due to artefacts (*n* = 12), or pre-existing NICM (*n* = 31). ROC curve analysis identified an optimal ECV cut-off of 29.5% for this population. Given its similarity to cut-offs from prior CT-ECV studies,^11^ the original thresholds were retained for subsequent analyses. Patients were stratified into four groups (ECV < 29.5% vs. ≥ 29.5%; no vs. present LIE) to evaluate the combined pattern of diffuse and focal fibrosis on delayed-phase CCT (*Figure 3*).

Baseline clinical characteristics stratified by LIE and ECV status are summarized in *Table 1*. The mean age was 69.4 ± 11.7 years, and 60% were male. LIE was present in 424 patients (35.1%), including 247 (20.4%) with an ischaemic pattern and 177 (14.7%) with a non-ischaemic pattern. The mean CT-ECV value was 30.2 ± 5.5%. Patients without LIE and with normal ECV had the lowest prevalence of hypertension, diabetes mellitus, and prior coronary revascularization. In contrast, those with LIE and elevated ECV exhibited the highest rates of hypertension, diabetes, and prior coronary revascularization, along with markedly elevated cardiac biomarkers (BNP and hs-cTnT) and reduced LVEF on echocardiography. These findings indicate that patients with LIE and elevated ECV experience more extensive myocardial injury and impaired systolic function. Furthermore, patients with LIE exhibited a higher prevalence of three-vessel disease and left anterior descending (LAD) coronary artery involvement compared with those without LIE.

**Table 1:** Baseline characteristics

	Overall	LIE (−) + normal ECV	LIE (−) + elevated ECV	LIE (+) + normal ECV	LIE (+) + elevated ECV	*P*-value
	(*n* = 1207)	(*n* = 485)	(*n* = 298)	(*n* = 132)	(*n* = 292)	
Male, *n* (%)	727 (60)	252 (52)	158 (53)	100 (76) ^ab^	217 (74) ^ab^	<0.001
Age, years	69.4 ± 11.7	68.5 ± 12.1	71.4 ± 11.5^a^	67.8 ± 11.6^b^	69.7 ± 10.8	0.002
Body mass index, kg/m^2^	23.6 ± 4.0	24.0 ± 3.7	23.0 ± 4.3^a^	24.1 ± 3.6^b^	23.2 ± 4.3	<0.001
Medical history						
Hypertension, *n* (%)	793 (66)	295 (61)	187 (63)	91 (69)	220 (75)^ab^	<0.001
Diabetes mellitus, *n* (%)	391 (32)	120 (25)	82 (28)	53 (40)^ab^	136 (47)^ab^	<0.001
Dyslipidaemia, *n* (%)	619 (51)	230 (47)	138 (46)	85 (64)^ab^	166 (57)^ab^	<0.001
Atrial fibrillation, *n* (%)	176 (15)	48 (10)	64 (21)^a^	16 (12)^b^	48 (16)^a^	<0.001
Previous revascularization						
PCI and/or CABG, *n* (%)	267 (22)	50 (10)	65 (22)^a^	41 (31)^ab^	111 (38)^ab^	<0.001
Prior myocardial infarction	116 (10)	5 (1)	4 (1)	34 (26)^b^	73 (25)^ab^	<0.001
Laboratory examination parameters						
Haematocrit, %	39.5 ± 5.8	40.5 ± 5.6	37.5 ± 5.6^a^	41.9 ± 5.6^ab^	38.9 ± 5.8^abc^	<0.001
hs-cTnT, ng/mL	0.012 [0.008–0.024]	0.010 [0.006–0.016]	0.014 [0.008–0.022] ^a^	0.013 [0.007–0.021] ^a^	0.022 [0.011–0.054] ^abc^	<0.001
BNP, pg/mL	36.8 [13.9–105.2]	20.2 [9.9–48.6]	44.3 [15.4–121.4] ^a^	33.1 [13.1–79.4] ^ab^	87.2 [30.9–257.7] ^abc^	<0.001
Sodium, mEq/L	139.9 ± 2.7	140.2 ± 2.4	139.9 ± 2.7	140.1 ± 2.6	139.5 ± 2.9^a^	<0.001
Potassium, mEq/L	4.4 ± 3.0	4.3 ± 0.4	4.2 ± 0.5	4.3 ± 0.5	4.3 ± 0.5	0.140
eGFR, mL/min/1.73 m^2^	62.1 ± 20.1	64.7 ± 18.0	59.4 ± 20.9^a^	64.3 ± 18.2	59.3 ± 22.8^a^	<0.001
Echocardiogram parameters						
LVEF, %	58.1 ± 10.0	61.4 ± 6.6	59.5 ± 9.1	57.6 ± 10.0	51.5 ± 12.1	<0.001
LVDd, mm	45.0 ± 6.9	43.8 ± 6.0	44.2 ± 7.0	45.6 ± 6.2^a^	47.3 ± 7.9^ab^	<0.001
LVDs, mm	30.5 ± 8.0	28.3 ± 6.2	29.4 ± 7.6	31.4 ± 6.7^a^	34.6 ± 9.5^abc^	<0.001
Intraventricular septal thickness, mm	10.4 ± 4.4	10.3 ± 6.6	10.1 ± 1.7	10.6 ± 1.8	10.9 ± 2.5	0.121
LV posterior wall thickness, mm	10.1 ± 1.8	9.8 ± 1.5	10.0 ± 1.6	10.2 ± 1.8	10.5 ± 2.3^ab^	<0.001
ECG						
QRS, mm	98.0 ± 20.2	95.9 ± 19.0	97.4 ± 21.5	101.3 ± 21.6^a^	100.5 ± 19.6^a^	<0.001
QT, mm	438.6 ± 31.3	432.6 ± 30.9	441.4 ± 34.1^a^	437.3 ± 26.0	446.0 ± 29.2^ac^	<0.001
CLBBB	31 (3)	10 (2)	15 (5)	3 (2)	3 (1)^b^	0.016
CT measurements						
Coronary artery disease, *n* (%)	617 (51)	180 (37)	148 (50)^a^	89 (67)^ab^	200 (68)^ab^	<0.001
1 vessel	234 (19)	92 (19)	63 (21)	25 (19)	54 (18)	0.848
2 vessels	148 (12)	45 (9)	31 (10)	19 (14)	53 (18)^ab^	0.002
3 vessels	230 (19)	42 (9)	53 (18)^a^	43 (33)^ab^	92 (32)^ab^	<0.001
CAD including LAD	527 (44)	142 (29)	131 (44)	77 (58)	177 (61)^a^	0.026
Left main coronary artery	44 (4)	13 (3)	7 (2)	6 (5)	18 (6)	0.496
ECV, %	30.2 ± 5.5	26.1 ± 2.2	32.9 ± 3.1^a^	26.5 ± 2.0^b^	35.9 ± 5.7^abc^	<0.001

Values are presented as the number of patients (%), mean ± standard deviation (SD), or median [interquartile range].

*Post hoc* pairwise comparisons were performed between individual subgroups, with appropriate correction for multiple comparisons.

LIE, late iodine enhancement; ECV, extracellular volume fraction; PCI, percutaneous coronary intervention; CABG, coronary artery bypass graft; hs-cTnT, high-sensitivity cardiac troponin T; BNP, B-type natriuretic peptide; eGFR, estimated glomerular filtration rate; LVEF, left ventricular ejection fraction; LVDd, left ventricular diastolic diameter; LVDs, left ventricular systolic diameter; LV, left ventricle; ECG, electrocardiogram; CLBBB, complete left bundle branch block; CT, computed tomography; CAD, coronary artery disease; LAD, left anterior descending coronary artery.

^a^ *P* < 0.05 vs. LIE (−) + normal ECV, ^b^*P* < 0.05 vs. LIE (−) + elevated ECV, ^c^*P* < 0.05 vs. LIE (+) + normal ECV.

Supplementary data online, *Table S2* compares elevated ECV with LIE presence by contrasting the no LIE/elevated ECV group with the LIE/normal ECV group. While the LIE cohort showed a higher prevalence of coronary risk factors, the elevated ECV cohort—characterized by renal impairment and advanced age—reflected the patients’ broader systemic condition.

### Primary outcome

During a mean follow-up of 26.0 ± 19.1 months, 149 first composite events occurred, including 112 all-cause deaths and 39 unplanned cardiovascular hospitalizations. Non-cardiac deaths were most often due to cancer (*n* = 68, 60.7%), followed by pneumonia (*n* = 9, 8.0%), cerebrovascular disease (*n* = 8, 7.1%), infections (*n* = 6, 5.4%), and other causes.

**Figure 4:**
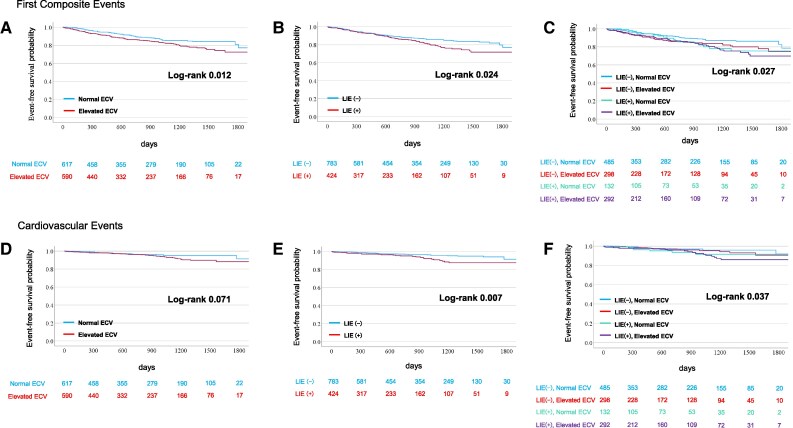
Kaplan–Meier cumulative incidence curves stratified by LIE and ECV. Kaplan–Meier curves for first composite events (*A*–*C*) and cardiovascular events (*D*–*F*) stratified by (*A* and *D*) composite LIE and ECV, (*B* and *E*) LIE (−) vs. LIE (+), and (*C* and *F*) normal vs. elevated ECV. LIE, late iodine enhancement; ECV, extracellular volume fraction.

Kaplan–Meier analysis showed that patients with elevated ECV had a significantly higher cumulative incidence of the composite endpoint than those with normal ECV (log-rank *P* = 0.012; *Figure 4A*). Similarly, patients with LIE had an increased risk compared with those without LIE (log-rank *P* = 0.024; *Figure 4B*). When stratified by LIE and ECV status, patients with both features had the highest event rates for the primary outcome (log-rank *P* = 0.027; *Figure 4C*).

Multivariable Cox regression confirmed that the combination of LIE and elevated ECV was independently associated with an increased risk of the primary outcome [adjusted hazard ratio (HR), 1.81; 95% confidence interval (CI), 1.17–2.81; *P* = 0.008; *Table 2*].

**Table 2:** Cox regression model proportional hazard models for first composite events and cardiovascular events

	Unadjusted		Adjusted	
	HR (95% CI)	*P*-value	HR (95% CI)	*P*-value
Cox regression analyses				
Primary outcome—all-cause death or unplanned cardiovascular hospitalization (149 events)				
Absence of LIE and normal ECV	Reference	Reference	Reference	Reference
Absence of LIE and elevated ECV	1.55 (1.02–2.37)	0.041	1.45 (0.95–2.23)	0.087
Presence of LIE and normal ECV	1.58 (0.91–2.73)	0.104	1.60 (0.91–2.82)	0.105
Presence of LIE and elevated ECV	1.84 (1.22–2.79)	0.004	1.81 (1.17–2.81)	0.008
Secondary outcome—cardiovascular events (51 events)				
Absence of LIE and normal ECV	Reference	Reference	Reference	Reference
Absence of LIE and elevated ECV	1.51 (0.69–3.30)	0.308	1.37 (0.62–3.03)	0.438
Presence of LIE and normal ECV	2.14 (0.85–5.37)	0.105	1.98 (0.77–5.13)	0.159
Presence of LIE and elevated ECV	2.67 (1.32–5.41)	0.006	2.40 (1.14–5.07)	0.022

HR, hazard ratio; LIE, late iodine enhancement; ECV, extracellular volume fraction.

### Secondary outcome

During follow-up, 12 cardiac deaths and 39 unplanned cardiovascular hospitalizations occurred (4.2% of the cohort), including 23 for HF and 16 for acute coronary syndrome. The time from CCT to revascularization for acute coronary syndrome was 17.0 ± 12.5 months.

The cumulative incidence of cardiac death or unplanned cardiovascular hospitalization was significantly higher in patients with LIE (log-rank *P* = 0.007; *Figure 4E*) but not in those with elevated ECV alone (log-rank *P* = 0.071; *Figure 4D*). However, the combination of LIE and elevated ECV demonstrated a synergistic effect in prognostic prediction (*Figure 4F*), with patients showing a significantly higher risk for the secondary outcome (adjusted HR, 2.40; 95% CI, 1.14–5.07; *P* = 0.022; *Table 2*).

### Prognostic impact according to LIE subtype

To further evaluate the prognostic value of LIE, the patients were stratified into three subgroups according to LIE subtype: none, ischaemic, and non-ischaemic.

Kaplan–Meier analysis revealed a significant difference in cardiovascular events among the three groups (log-rank *P* = 0.021; Supplementary data online, *Figure S1*). In the unadjusted Cox proportional hazards analysis, ischaemic LIE was associated with cardiovascular events, whereas non-ischaemic LIE was associated with the first composite events. After adjusting for baseline covariates, ischaemic LIE exhibited adjusted HRs of 1.33 (95% CI, 0.85–2.07; *P* = 0.207) and 1.78 (95% CI, 0.87–3.63; *P* = 0.113) for the first composite and cardiovascular events, respectively. In contrast, non-ischaemic LIE exhibited adjusted HRs of 1.57 (95% CI, 1.03–2.41; *P* = 0.036) and 2.02 (95% CI, 0.96–4.24; *P* = 0.036) for the first composite and cardiovascular events, respectively (see Supplementary data online, *Table S3*). These findings suggest that non-ischaemic LIE confers a stronger prognostic impact than ischaemic LIE.

### Identification of cardiomyopathy by CCT

**Figure 5:**
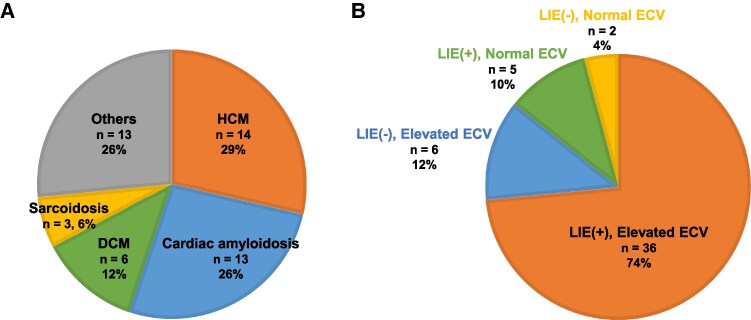
Percentage of patients diagnosed with cardiomyopathy by CCT. (*A*) Types of NICM in patients diagnosed with NICM by CCT. (*B*) Classification of patients with cardiomyopathy by combined LIE presence and normal or elevated ECV. CCT, cardiac computed tomography; NICM, non-ischaemic cardiomyopathy; LIE, late iodine enhancement; ECV, extracellular volume fraction.

Of the 1207 patients evaluated, 49 were newly diagnosed with NICM using CMR, 99mTc-PYP scintigraphy, and endocardial biopsy. The most common aetiologies were HCM (*n* = 14, 28.5%), cardiac amyloidosis (*n* = 13, 26.5%), and DCM (*n* = 6, 12.2%) (*Figure 5A*).

Subgroup analysis showed that most NICM cases (*n* = 36, 73.4%) belonged to the group with LIE and elevated ECV, followed by elevated ECV without LIE (*n* = 6, 12.2%) and LIE without elevated ECV (*n* = 5, 10.2%) (*Figure 5B*). Supplementary data online, *Table S4* compares patients with and without NICM in the LIE and elevated ECV group. Although coronary artery stenosis was identified by CCT in 17 patients within the NICM cohort, myocardial ischaemia was excluded by coronary angiography and myocardial scintigraphy. These patients often had higher BNP and hs-cTnT levels and lower LVEF on echocardiography. The NICM group also had significantly greater LV wall thickness and elevated ECV.

### Impact of NICM on study outcomes

**Figure 6:**
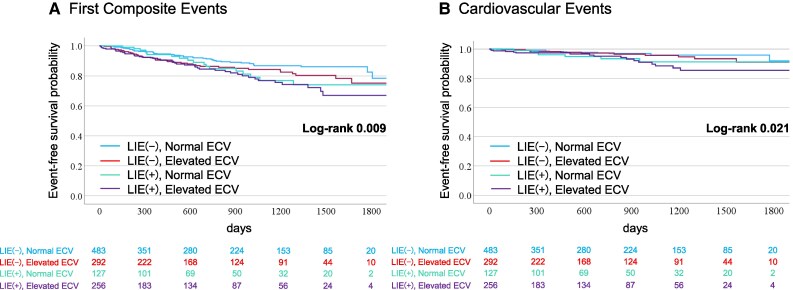
Kaplan–Meier survival curves stratified by composite LIE and ECV for first composite and cardiovascular events, excluding cardiomyopathy diagnosed by CCT. (*A*) First composite events and (*B*) cardiovascular events, excluding patients with NICM diagnosed by CCT. LIE, late iodine enhancement; ECV, extracellular volume fraction; NICM, non-ischaemic cardiomyopathy; CCT, cardiac computed tomography.

Because cardiomyopathies such as HCM and cardiac amyloidosis (*Figure 5*) may have influenced the outcomes, these cases were excluded and reanalysed using Kaplan–Meier and Cox regression. Patients with LIE and elevated ECV continued to show higher risk for the primary and secondary outcomes (*Figure 6*). The adjusted HR for the primary outcome remained significant (HR, 2.04; 95% CI, 1.30–3.19; *P* = 0.002; *Table 3*). The secondary outcome risk was also increased (adjusted HR, 2.57; 95% CI, 1.18–5.60; *P* = 0.018).

**Table 3:** Cox regression model proportional hazard models for first composite events and cardiovascular events excluding cardiomyopathy diagnosed by CCT

	Unadjusted		Adjusted	
	HR (95% CI)	*P*-value	HR (95% CI)	*P*-value
Cox regression analyses				
Primary outcome—all-cause death or unplanned cardiovascular hospitalization (149 events)				
Absence of LIE and normal ECV	Reference	Reference	Reference	Reference
Absence of LIE and elevated ECV	1.51 (0.98–2.31)	0.060	1.43 (0.93–2.21)	0.104
Presence of LIE and normal ECV	1.65 (0.95–2.85)	0.075	1.69 (0.96–2.99)	0.071
Presence of LIE and elevated ECV	2.05 (1.34–3.13)	<0.001	2.04 (1.30–3.19)	0.002
Secondary outcome—cardiovascular events (51 events)				
Absence of LIE and normal ECV	Reference	Reference	Reference	Reference
Absence of LIE and elevated ECV	1.41 (0.63–3.14)	0.403	1.30 (0.58–2.92)	0.531
Presence of LIE and normal ECV	2.24 (0.89–5.61)	0.087	2.14 (0.82–5.57)	0.121
Presence of LIE and elevated ECV	2.86 (1.39–5.90)	0.004	2.57 (1.18–5.60)	0.018

CCT, cardiac computed tomography; HR, hazard ratio; LIE, late iodine enhancement; ECV, extracellular volume fraction.

## Discussion

LIE, similar to LGE on CMR, is valuable for diagnosing cardiomyopathy and identifying myocarditis lesions, providing visual assessment of focal myocardial injury.^12,13^ However, it may underestimate diffuse myocardial injury. In contrast, ECV quantifies diffuse myocardial injury resulting from myocardial fibrosis, low-grade oedema, or reactive fibrosis.^14^ Therefore, LIE and ECV serve as complementary biomarkers, each evaluating myocardial injury from a distinct perspective. In this study, we combined LIE and ECV values to investigate their association with all-cause mortality and cardiovascular events in patients undergoing CCT for coronary artery evaluation.

CT-ECV has gained attention as a marker of fibrosis in various cardiac diseases, and in this study, event incidence was significantly higher in the elevated ECV group (≥29.5%).^9,21^ A recent meta-analysis of patients with severe AS reported that the CT-ECV threshold is ∼30% and that elevated ECV is associated with more than a fourfold increase in cardiovascular event risk compared with normal ECV.^9^ The ECV threshold in this study was derived from ROC curve analysis, aligning with previous evidence that healthy myocardium typically exhibits ECV values of 23–28%.^11,22,23^

LIE, like LGE on CMR, can detect myocardial injury not only in ischaemia but also in myocarditis and NICM.^24^ In LGE, the presence and extent of delayed enhancement are associated with HF and fatal arrhythmia, and LIE is also considered to reflect fibrosis and scarring.^25,26^ Patients with LIE exhibited a higher prevalence of three-vessel disease and LAD involvement, suggesting that LIE tended to occur in individuals with more extensive CAD. Previous studies have reported that LIE positivity among patients with coronary artery disease is associated with worse outcomes compared with those of patients without LIE.^27^ In this study, non-ischaemic LIE was independently associated with adverse outcomes, indicating that it represents a strong marker of myocardial injury and provides incremental prognostic information beyond coronary anatomical characteristics. In terms of cardiac death and unplanned cardiovascular hospitalization, event frequency was significantly higher in the LIE group, whereas elevated ECV alone was not significantly associated with these outcomes. This may be explained by the fact that ECV can be elevated in conditions, such as hypertension and diabetes, thereby identifying early myocardial damage.^28–30^

The most noteworthy finding of this study is that event rates were significantly higher in the LIE and elevated ECV group than those in the no LIE and normal ECV group for primary and secondary outcomes. Elevated ECV has been associated with a high risk of HF in patients with LGE on CMR,^31^ and our findings similarly suggest a synergistic relationship: patients with LIE and elevated ECV exhibited the greatest risk of cardiovascular events. These results indicate that LIE and ECV each contribute to prognostic assessment as indicators of fibrosis and myocardial structural abnormalities, and that their combination may enable novel risk stratification that complements conventional functional and anatomical markers.

In infiltrative cardiomyopathies, such as cardiac amyloidosis and in AS, ECV has demonstrated value for prognostic evaluation and personalized treatment planning.^32–34^ CT-ECV has also been reported to incidentally identify cardiomyopathy.^35^ In this study, no patients had severe AS, and 49 patients were diagnosed with cardiomyopathy. After excluding these cases, patients with LIE and elevated ECV continued to show high event risk, suggesting that the prognostic utility of the combined index persists even when known cardiomyopathy is absent in CCT performed for CAD evaluation. Furthermore, nearly 80% of diagnosed cardiomyopathy cases fell within the LIE and elevated ECV group, underscoring the value of cardiac CT not only for risk stratification but also for incidental cardiomyopathy detection.

### Study limitations

First, this was a single-centre, retrospective study with a relatively small number of cardiovascular events, potentially limiting the precision of risk estimation. Validation in a similar cohort would strengthen these findings. Nevertheless, this represents the first cohort study assessing LIE and CT-ECV in a substantial number of patients undergoing CCT for coronary artery evaluation. Larger prospective studies are required to validate these results.

Second, ECV was measured by placing the ROIs in the septal segment of mid-ventricular short axis. Previous studies have reported that septal segments exhibit less bias in ECV quantification compared with non-septal segments, and the pooled correlation values for septal segments are significantly higher than those for non-septal segments.^8^ However, ECV was derived from a single mid-ventricular short-axis slice, which may not fully capture global myocardial tissue characteristics, potentially introducing variability and weakening associations with cardiovascular events. Lastly, delayed-phase images were acquired 7 min after the first contrast injection during diastole. ECV during diastole is 2–3% lower than during systole;^36^ however, the optimal timing for ECV measurement remains unclear.^37^ Protocols for CT-ECV vary among institutions.

## Conclusion

In this cohort of patients undergoing CCT for coronary artery evaluation, LIE and elevated ECV were each associated with cardiovascular events. Combining these measures may improve risk stratification beyond conventional cardiac assessments.

## Supplementary Material

jeag018_Supplementary_Data

## Data Availability

The data underlying this article will be shared upon reasonable request from the corresponding authors.
