# Supplementary material for: Does adding a delayed phase to cardiac computed tomography for coronary artery evaluation have prognostic value?
Source: Eur Heart J Cardiovasc Imaging. 2026 Jan 22;27(5):969–80. doi: 10.1093/ehjci/jeag018 (PMC13128271; doi:10.1093/ehjci/jeag018)
Supplement: jeag018_Supplementary_Data [file jeag018_supplementary_data.docx]

Supplemental Table S1. Number of participants with missing data for baseline characteristics

| Baseline Characteristics | LIE (-)  + Normal ECV | LIE (-)  + Elevated ECV | LIE (+)  + Normal ECV | LIE (+)  + Elevated ECV |
| --- | --- | --- | --- | --- |
|  | (n=485) | (n=298) | (n=132) | (n=292) |
| hs-cTnT | 15 (3) | 8 (3) | 4 (3) | 5 (2) |
| BNP | 28 (6) | 10 (3) | 8 (6) | 5 (2) |
| Sodium | 8 (2) | 5 (2) | 1 (1) | 0 (0) |
| Potassium | 8 (2) | 5 (2) | 1 (1) | 0 (0) |
| LVEF | 3 (1) | 2 (1) | 2 (2) | 0 (0) |
| LVDd | 3 (1) | 2 (1) | 2 (2) | 0 (0) |
| LVDs | 3 (1) | 2 (1) | 2 (2) | 0 (0) |
| Intraventricular septal thickness | 3 (1) | 2 (1) | 2 (2) | 0 (0) |
| LV posterior wall thickness | 3 (1) | 2 (1) | 2 (2) | 0 (0) |
| QRS | 15 (3) | 5 (2) | 4 (3) | 2 (1) |
| QT | 15 (3) | 5 (2) | 4 (3) | 2 (1) |
| CLBBB | 15 (3) | 5 (2) | 4 (3) | 2 (1) |

LIE, late iodine enhancement; ECV, extracellular volume fraction; hs-cTnT, high-sensitivity cardiac troponin T; BNP, B-type natriuretic peptide; LVEF, left ventricular ejection fraction; LVDd, left ventricular diastolic diameter; LVDs, left ventricular systolic diameter; LV, left ventricle; CLBBB, complete left bundle branch block

Supplemental Table S2. Comparison of elevated ECV and present LIE

|  | LIE (-)  + Elevated ECV  (n=298) | LIE (+)  + Normal ECV  (n=132) | P value |
| --- | --- | --- | --- |
| Male, n (%) | 158 (53) | 100 (76) | <0.001 |
| Age, years | 71.4±11.5 | 67.8±11.6 | 0.003 |
| Body mass index, kg/m^2^ | 23.0±4.3 | 24.1±3.6 | 0.006 |
| Hypertension, n (%) | 187 (63) | 91 (69) | 0.216 |
| Diabetes mellitus, n (%) | 82 (28) | 53 (40) | 0.009 |
| Dyslipidaemia, n (%) | 138 (46) | 85 (64) | <0.001 |
| Atrial fibrillation, n (%) | 64 (21) | 16 (12) | 0.021 |
| PCI and/or CABG | 65 (22) | 41 (31) | 0.040 |
| Haematocrit, % | 37.5±5.6 | 41.9±5.6 | <0.001 |
| hs-cTnT, ng/mL | 0.014 [0.008–0.022] | 0.013 [0.007–0.021] | 0.258 |
| BNP, pg/mL | 44.3 [15.4–121.4] | 33.1 [13.1–79.4] | 0.026 |
| Sodium, mEq/L | 139.9±2.7 | 140.1±2.6 | 0.552 |
| Potassium, mEq/L | 4.2±0.5 | 4.3±0.5 | 0.531 |
| eGFR, mL/min /1.73m^2^ | 59.4±20.9 | 64.3±18.2 | 0.023 |
| LVEF, % | 59.5±9.1 | 57.6±10.0 | 0.067 |
| LVDd, mm | 44.2±7.0 | 45.6±6.2 | 0.048 |
| LVDs, mm | 29.4±7.6 | 31.4±6.7 | 0.012 |
| IVST, mm | 10.1±1.7 | 10.6±1.8 | 0.003 |
| LV PWT, mm | 10.0±1.6 | 10.2±1.8 | 0.142 |
| QRS, mm | 97.4±21.5 | 101.3±21.6 | 0.094 |
| QT, mm | 441.4±34.1 | 437.3±26.0 | 0.234 |
| Extracellular volume, % | 32.9±3.1 | 26.5±4.0 | <0.001 |
| Coronary artery disease, n (%) | 148 (50) | 89 (67) | 0.035 |

Values are presented as the number of patients (%), mean ± standard deviation (SD), or median (interquartile range).

LIE, late iodine enhancement; ECV, extracellular volume fraction; PCI, percutaneous coronary intervention; CABG, coronary artery bypass graft; hs-cTnT, high-sensitivity cardiac troponin T; BNP, B-type natriuretic peptide; eGFR, estimated glomerular filtration rate; LVEF, left ventricular ejection fraction; LVDd, left ventricular diastolic diameter; LVDs, left ventricular systolic diameter; LV, left ventricle; IVST, Intraventricular septal thickness; PWT, posterior wall thickness

Supplemental Table S3. Cox regression model proportional hazard models for first composite events and cardiovascular events according to LIE subtype

|  | Unadjusted |  | Adjusted |  |
| --- | --- | --- | --- | --- |
|  | HR (95% CI) | P value | HR (95% CI) | P value |
| Cox regression analyses | | | | |
| Primary outcome - all-cause death or unplanned cardiovascular hospitalization (149 events) | | | | |
| No LIE | Reference | Reference | Reference | Reference |
| Ischemic LIE | 1.36 (0.92-2.03) | 0.126 | 1.33 (0.85-2.07) | 0.207 |
| Nonischemic LIE | 1.59 (1.04-2.42) | 0.032 | 1.57 (1.03-2.41) | 0.036 |
| Secondary outcome - cardiovascular events (51 events) | | | | |
| No LIE | Reference | Reference | Reference | Reference |
| Ischemic LIE | 2.28 (1.21-4.27) | 0.010 | 1.78 (0.87-3.63) | 0.113 |
| Nonischemic LIE | 1.89 (0.91-3.93) | 0.090 | 2.02 (0.96-4.24) | 0.063 |

HR, hazard ratio; LIE, late iodine enhancement

Supplemental Table S4. Comparison of patients with and without nonischemic cardiomyopathy in the LIE and elevated ECV group

|  | All  (n = 292) | NICM (-)  (n = 256) | | NICM (+)  (n = 36) | P value |
| --- | --- | --- | --- | --- | --- |
| Demographics |  |  | |  |  |
| Male, n (%) | 217 (74) | 192 (25) | | 25 (70) | 0.475 |
| Age, years | 69.7±10.8 | 70.2±9.9 | | 65.8±15.1 | 0.094 |
| Body mass index, kg/m^2^ | 23.2±4.3 | 23.0±4.2 | | 25.0±4.8 | 0.011 |
| Medical history |  | |  |  |  |
| Hypertension, n (%) | 220 (75) | 195 (76) | | 25 (70) | 0.381 |
| Diabetes mellitus, n (%) | 136 (47) | 125 (49) | | 11 (31) | 0.040 |
| Dyslipidaemia, n (%) | 166 (57) | 151 (59) | | 15 (42) | 0.049 |
| Atrial fibrillation, n (%) | 48 (16) | 42 (16) | | 6 (17) | 0.969 |
| Previous revascularisation |  |  | |  |  |
| PCI and/or CABG | 111 (38) | 110 (43) | | 1 (3) | <0.001 |
| Laboratory examination parameters |  |  | |  |  |
| Haematocrit, % | 38.9±5.8 | 38.6±5.9 | | 41.5±3.7 | <0.001 |
| hs-cTnT, ng/mL | 0.022 [0.011–0.054] | 0.022 [0.011-0.055] | | 0.026 [0.010-0.053] | 0.887 |
| BNP, pg/mL | 87.2 [30.9–257.7] | 88.0 [31.5-256.8] | | 64.3 [22.0-265.3] | 0.574 |
| Sodium, mEq/L | 139.5±2.9 | 139.3±3.0 | | 140.4±2.1 | 0.012 |
| Potassium, mEq/L | 4.3±0.5 | 4.3±0.5 | | 4.1±0.5 | 0.084 |
| eGFR, mL/min /1.73m^2^ | 59.3±22.8 | 58.4±23.5 | | 65.6±15.5 | 0.077 |
| Echocardiogram parameters |  |  | |  |  |
| LVEF, % | 51.5±12.1 | 51.7±12.3 | | 50.0±10.9 | 0.428 |
| LVDd, mm | 47.3±7.9 | 47.6±8.0 | | 45.2±7.2 | 0.091 |
| LVDs, mm | 34.6±9.5 | 34.7±9.7 | | 34.0±8.1 | 0.663 |
| Intraventricular septal thickness, mm | 10.9±2.5 | 10.7±2.1 | | 12.5±4.0 | 0.009 |
| LV posterior wall thickness, mm | 10.5±2.3 | 10.3±1.9 | | 12.1±3.9 | 0.010 |
| ECG |  |  | |  |  |
| QRS, mm | 100.5±19.6 | 100.6±19.4 | | 100.1±21.6 | 0.891 |
| QT, mm | 446.0±29.2 | 445.3±28.9 | | 450.5±31.4 | 0.320 |
| CT measurements |  |  | |  |  |
| Extracellular volume, % | 35.9±5.7 | 33.8±4.3 | | 39.3±11.1 | <0.001 |
| Coronary artery disease, n (%) | 200 (68) | 182 (71) | | 18 (50) | 0.010 |

Values are presented as the number of patients (%), mean ± standard deviation (SD), or median (interquartile range).

LIE, late iodine enhancement; ECV, extracellular volume fraction; NICM, nonischemic cardiomyopathy; PCI, percutaneous coronary intervention; CABG, coronary artery bypass graft; hs-cTnT, high-sensitivity cardiac troponin T; BNP, B-type natriuretic peptide; eGFR, estimated glomerular filtration rate; LVEF, left ventricular ejection fraction; LVDd, left ventricular diastolic diameter; LVDs, left ventricular systolic diameter; LV, left ventricle

Supplemental Figure S1. Kaplan–Meier survival curves stratified by LIE subtype (none, ischemic, and nonischemic)


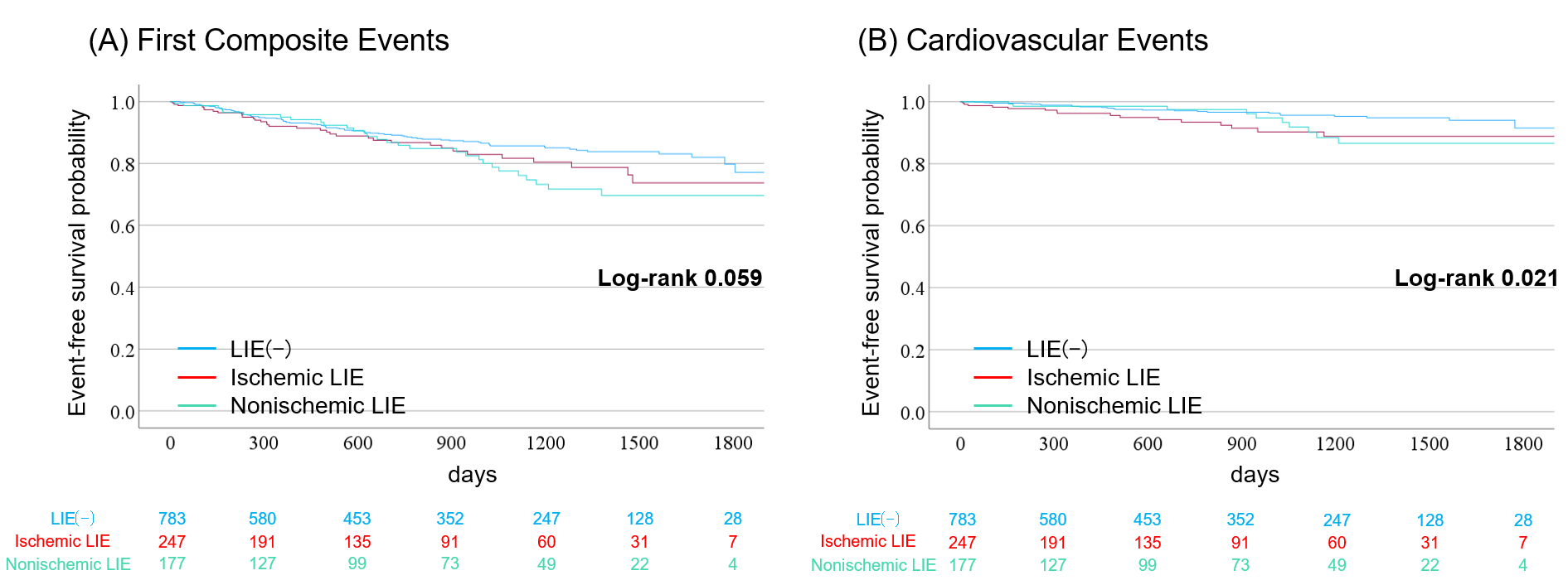


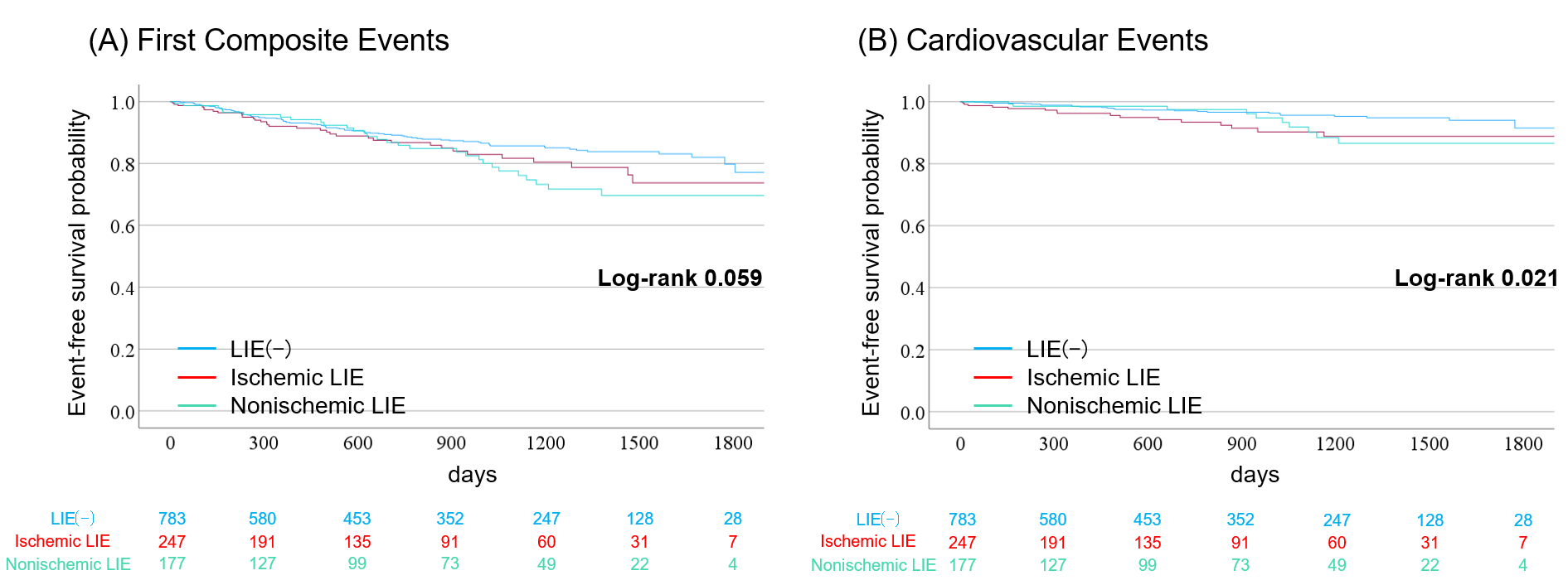


Patients were stratified into three groups: none, ischemic, and nonischemic LIE.

LIE, late iodine enhancement
